# Clerical Encouragement of Quackery

**Published:** 1841-09

**Authors:** 


### CLERICAL ENCOURAGEMENT OF QUACKERY.

We can scarcely open a newspaper, without meeting with the advertisement of one or more quack medicines, recommended and avouched by clergymen. Now such is the confidence of the mass of the people in their spiritual pastors, that these certificates have in them a power, even greater than the forged testimonials of eminent, deceased physicians, so often seen appended to the same advertisements. Such being the case, we would respectfully ask our clerical friends, to whom we attribute no bad motive in this matter, whether they have ever reflected on the mischief they do to the community, by these recommendations? Do they not know, that if a nostrum be *inert*, a reliance upon it may destroy life—if *active*, that while it may relieve or even cure a few, it will kill many more? We would charitably believe, that most of these certificates are given, without due reflection. The majority of them are for cough mixtures, balsams, bolusses or lozenges, which are presented as infallible remedies, without reference to the nature of the disease in the lungs, by which the cough is produced. But the diseases of the lungs are of various kinds—requiring different modes of treatment—and what may cure one patient will destroy another. If a clergyman, then, has seen a quack medicine relieve one individual, he is not justified in generalizing, and commending it to all who may, from the coincidence of a single symptom, fancy themselves in the same condition.

Medicine is an inductive science, the basis of which is a knowledge of the structure and functions of the human body. He who builds on this foundation, rests his superstructure on a rock—all others build on sand. How many of our clergyman, understand anatomy and physiology, beyond Dr. Paley's Natural Theology? We suspect, very few. We would ask these respected brethren, what they mean by orthodoxy? Is it not a full acquaintance with the letter and spirit of the Bible, and a faithful adherence to both? Now medicine, so to speak, has *its* orthodoxy, which consists in a profound knowledge of the principles of the science, and a reliance on them to guide us in practice, as the divine relies on the doctrines of the Bible to guide and govern him in preaching. If some ignorant layman, but superficially acquainted with that divine revelation and unimbued with its spirit, were to advertise a new exposition of its doctrines—a sort of patent mode of securing Heaven, what would our clerical friends say, if physicians who had never made the Bible a study, were to certify to the truth and efficacy of such a pretended discovery? They would, undoubtedly warn the people to beware. It would be a dereliction of duty for them to remain silent; and we, on the other hand, feel, that duty in reference to the health and temporal welfare of the community, commands *us* to speak out, in words of warning to the people, and of rebuke to such of their spiritual leaders, as travel out of their profession, to enlist under the banner of quackery in another.

